# Multi-omics reveals effects of diet FNDF/starch level on growth performance and rumen development of Hu sheep

**DOI:** 10.3389/fmicb.2025.1601950

**Published:** 2025-08-05

**Authors:** Haibi Zhao, Jiqing Wang, Zhiyun Hao, Pengfei Yin, Shanglong Wang, Yanli Guo, Chunyan Ren

**Affiliations:** College of Animal Science and Technology, Gansu Agricultural University, Lanzhou, China

**Keywords:** Hu sheep, diet FNDF/starch, rumen, multi-omics, growth performance

## Abstract

To maximize the utilization of nutritional components in diet to enhance the growth performance of Hu sheep, this study investigates the effects of varying forage neutral detergent fiber (FNDF) to starch ratio levels in the diet on the rumen microbial flora, metabolites and expression in the rumen epithelium through sequencing techniques involving microbiomes, metabolomics and transcriptomes. Thirty-six male Hu sheep (2 months old) with similar weight [(10 ± 0.5) kg] were randomly divided into four groups of nine sheep each, and were divided into groups A (FNDF/starch = 0), B (FNDF/starch = 0.23), C (FNDF/starch = 0.56) and D (FNDF/starch = 1.10) with different FNDF/starch of pelleted rations, respectively. The results indicated that average daily gain (ADG) and average daily feed intake (ADFI) of group B, C and D were significantly higher than group A (*p* < 0.05); the feed conversion ratio (FCR) of group B was significantly higher than other groups (*p* < 0.05) and the height of rumen papillae in group B and C was significantly higher than in group A (*p* < 0.05). Species annotation results of microorganisms were found that the presence of 22 phyla, 33 classes, 62 orders, 120 families, 245 genera and 341 species. Among them, *Prevotella_7* (20.170%) and *Succinivibrionaceae_UCG_001* (12.28%) were the dominant bacteria at the genus; Bacteroidota (36.66%), Firmicutes (33.06%) and Proteobacteria (25.39%) were dominant at the phylum. A total of 3,907 metabolites were annotated by metabolomic analysis of the rumen content samples and the differential metabolites were mainly enriched in amino acid metabolism, cofactor and vitamin metabolism and lipid metabolism. Rumen epithelial transcriptome sequencing analysis identified 825 (A vs. B), 355 (A vs. C), 818 (A vs. D), 204 (B vs. C), 718 (B vs. D) and 199 (C vs. D) differentially expressed genes (DEGs). DEGs were mainly enriched in pathways related to amino acid metabolism, vitamins metabolism and signaling, etc. Notably, during histidine metabolism, thiamine in the rumen decreased with increasing FNDF/starch levels, while the expression level of the TPK1 in the rumen epithelium increased with rising FNDF/starch levels. In conclusion, diet FNDF/starch levels have a significant effect on growth performance and healthy rumen development of Hu sheep.

## Introduction

Forage and concentrated feed are, respectively, the main sources of neutral detergent fiber (NDF) and starch in the diet (Piantoni et al., 2015). Adjusting the proportion of forage and concentrate in the diet can effectively change the levels of NDF and starch in the diet, and the levels of NDF and starch are closely related to rumen fermentation and production performance of lambs. For example, a diet high in starch and low in NDF accelerates the rumen fermentation rate, resulting in the production of a large amount of volatile fatty acids (VFA) in the short term and reducing the rumen pH value. However, this may lead to metabolic disorders, such as rumen acidosis (Wallace et al., 2001; Colombatto et al., 2003; Zhang et al., 2018). On the contrary, feeding a low-starch diet may lead to insufficient fermentable energy in the rumen, disrupt the functions of microorganisms, reduce the absorption and barrier capacity of rumen epithelium, and impair the utilization of nutrients (Mulakala et al., 2023). Furthermore, studies have shown that diets with high NDF produce a higher molar ratio of acetate (acetic acid-type fermentation), while starch-based diets produce a higher molar ratio of propionate (propionic acid-type fermentation) (Suárez et al., 2006; Detmann et al., 2014; EbnAli et al., 2016). Changes in fermentation type directly affect the composition and abundance of microorganisms (Su et al., 2022). Research has shown that as starch content increases, the abundance of *Streptococcus bovis* in lambs rises, while the abundance of *Butyrivibrio* decreases, leading to a reduction in total bacterial counts (Guo et al., 2021). Increasing the NDF level in the diet can enhance the abundance of fiber-digesting bacteria in the rumen; for example, the addition of oat hay to sheep diets can increase the abundance of Bacteroidetes (Xie et al., 2020; Liu et al., 2016; An et al., 2020). Furthermore, the interaction between microorganisms and their hosts is mediated by the interaction of metabolites between the host and the bacteria (Su et al., 2022; Temmar et al., 2021; Ren et al., 2018; Vi et al., 2004). Rumen epithelium is the channel connecting rumen contents and rumen tissues, allowing microbial metabolites to act on the host (Shabat et al., 2016; Zhang R. et al., 2021). At present, many studies have reported the relationship between the composition of rumen microorganisms and its metabolites in ruminants and the composition of their diets, indicating that feed affects the composition of rumen microbiota and its metabolites and the interaction between microbial metabolites and the host affects the expression of host genes, further promoting the growth and development of animals (Zhang et al., 2022; Zhao et al., 2023; Torres Manno et al., 2023). For instance, the anaerobic environment in the rumen and the fermentation activity of rumen microorganisms enable plant substances to ferment into metabolic end products, such as VFAs. VFAs can be metabolized, absorbed and transported through the rumen epithelium, interacting with various physiological functions of the host (Steele et al., 2016). This interaction is ultimately reflected in the growth and development of the host (Ren et al., 2023; Malmuthuge et al., 2019). However, at present, it is still rare to combine the dietary FNDF/starch level with the changes of rumen microorganisms to study the effect of dietary FNDF/starch level on the host. Therefore, in this study, microbiomics, non-targeted metabolomics and transcriptomics were used to explore the effects of dietary FNDF/starch levels on rumen microorganisms and their metabolites as well as the host, and to further identify the optimal balance between NDF and starch in the diet. This is crucial for enhancing the nutrient utilization rate and production capacity of ruminants.

## Materials and methods

### Experimental animals and sample collection

All animal experiments received approval from the Animal Ethics Committee of Gansu Agricultural University, Gansu, China (approval number: GSAU-Eth-Ast-2021-008). Thirty-six weaned male Hu sheep lambs with similar ages [(60 ± 5) days] and body weights [(10.0 ± 0.5) kg] were selected and randomly divided into four groups, with 9 lambs in each group. These groups were designated as group A (FNDF/starch = 0), group B (FNDF/starch = 0.23), group C (FNDF/starch = 0.56), and group D (FNDF/starch = 1.12). The diet formulation was in accordance with the “Feeding Standards for Meat Sheep and Goats (NY/T816–2021).” Feed ingredients were crushed and then pelletized using a ring die (with a temperature of 84–86°C; a die size of 8.0 mm, a compression ratio of 1:5, and a pellet diameter of 4 mm) to produce pellet feed. The composition and nutritional components of the diet were detailed in Table 1. The experimental period lasted for 60 days, including 10 days of pre-feeding and 50 days of formal feeding. On the first day of the experiment and every 10 days thereafter at 8:00 a.m. (before feeding), the Hu sheep were weighed. Feeding was carried out at 8:00 and 16:00 every day, and the feeding amounts were recorded. The remaining feed from the previous day was weighed before feeding at 8:00 a.m. each morning. The Feed Conversion Ratio (FCR) was calculated based on the ratio of total feed consumption to net body weight gain post-experiment. Prior to slaughter, Hu sheep were fasted for 24 h and subjected to water deprivation for 2 h. Six experimental sheep per group were electrically stunned followed by cervical exsanguination. Post-slaughter, rumen abdominal tissue was rapidly excised and rinsed thoroughly with sterile physiological saline to remove surface contaminants. The rumen epithelium was isolated, sectioned into ~1 cm^2^ samples, and stored in pre-chilled cryovials under liquid nitrogen. Concurrently, rumen contents were collected and filtered through four layers of sterile gauze to remove coarse particles. The filtrate was aliquoted into cryotubes and temporarily stored in liquid nitrogen for subsequent analysis. All samples were transported to the laboratory and archived at −80°C for downstream analyses, including rumen fermentation parameters, high-throughput 16S rRNA gene sequencing, metabolomics, and transcriptomics. Additionally, 1 cm^3^ whole rumen tissue samples were collected and fixed in 10% neutral-buffered formaldehyde solution for histological sectioning and microscopic observation. Tissue section preparation and measurement were performed according to a previously established protocol (Zhao et al., 2023). Fixed rumen abdominal tissues were processed through an automatic dehydrator: sequential dehydration in 75% ethanol (4 h), 85% ethanol (2 h), 95% ethanol (1 h), and 100% ethanol (0.5 h, 4 times); clearing in xylene (10 min, 2 times); and infiltration in paraffin (6 h). Tissues were then trimmed, embedded, sectioned, dewaxed, and stained with hematoxylin–eosin (HE). Hematoxylin stained cell nuclei blue-purple, while eosin stained cytoplasm pink. Following staining, sections were dehydrated with ethanol, cleared with xylene, sealed with neutral gum, and air-dried. Images were captured using an upright optical microscope (Nikon, Japan). For each sample, 10–15 sites were selected based on morphological characteristics, and papillary height, width, and basal layer thickness were measured using Image Pro Plus software (Media Cybernetics, Bethesda, MD, United States). Rumen fermentation parameters were determined as described (Liu et al., 2020). VFAs were analyzed via gas chromatography (GC-2010 Plus, Shimadzu, Kyoto, Japan) using the internal standard method with 2-ethylbutyric acid (2-EB). Concentration calculations involved: (1) determining the peak area ratio of target VFAs to 2-EB; (2) substituting ratios into calibration curve equations to obtain concentration ratios; and (3) calculating sample VFA concentrations. Rumen fluid pH was measured using a PHS-3C pH meter (Shanghai Precision Scientific Instruments, China).

**Table 1:** The composition and nutrient levels of diets (DM basis) %.

Item	Group			
	A	B	C	D
Composition (%)				
Corn	60	48.7	38	26.5
Soybean meal	27.7	25.3	22.2	20.13
Wheat bran	8	8	8	8
Alfalfa hay	0	6.9	16	21.8
Oat grass	0	7	12	20
Limestone	2.22	2	1.67	1.45
CaHPO_4_	0.73	0.75	0.78	0.77
Salt	0.35	0.35	0.35	0.35
Premix^1^	1	1	1	1
Total	100	100	100	100
Nutrient^2^				
DM	83.21	83.73	84.28	84.85
CF	3.43	7.99	12.70	17.18
NDF	12.60	19.22	25.81	32.44
ADF	5.33	9.94	14.61	19.19
CP	19.04	18.79	18.46	18.26
Ca	1.12	1.16	1.17	1.18
P	0.66	0.64	0.62	0.60
FNDF	0.00	8.00	16.00	23.99
Starch	41.95	35.02	28.38	21.36
FNDF/Starch	0.00	0.23	0.56	1.12

^1^The premix provided the following per kg of diets: VA 940 IU, VD 111 IU, VE 20 IU, VB12 0.02 mg, Cu 8 mg, Fe 25 mg, Mn 40 mg, Zn 40 mg, I 0.3 mg, Se 0.20 mg, S 200 mg, Co 0.1 mg. ^2^DM, CF, NDF, ADF, CP, Ca, P and were starch measured values, while FNDF is calculated based on the composition of the diets.

### Microbiological analysis of rumen contents

The genomic DNA of rumen contents was extracted using the TGuide S96 Stool DNA Kit (DP812, Tiangen Biotech, Beijing, China) according to the manufacturer’s protocol. DNA quality and quantity were assessed by 1.8% agarose gel electrophoresis, and concentration/purity were determined with a NanoDrop 2000 spectrophotometer (Sloan et al., 2021) (Thermo Scientific, Wilmington, DE, United States). The V1-V9 hypervariable regions of the 16S rRNA gene were amplified using primers 27F (5’-AGRGTTTGATYNTGGCTCAG-3′) and 1492R (5’-TASGGHTACCTTGTTASGACTT-3′), with sample-specific PacBio barcodes appended to both primers for multiplexed sequencing. The genomic DNA of the rumen contents was extracted using TGuide S96 Stool DNA Kit (DP812, Tiangen Biotech (Beijing) Co., Ltd.) according to manufacturer’s instructions. The quality and quantity of the extracted DNA were examined using electrophoresis on a 1.8% agarose gel, and DNA concentration and purity were determined with NanoDrop 2000 UV–Vis spectrophotometer (Thermo Scientific, Wilmington, United States). The V1-V9 hypervariable regions of the 16S rRNA gene were amplified using primers (27F: 5’-AGRGTTTGATYNTGGCTCAG-3′; 1492R: 5’-TASGGHTACCTTGTTASGACTT-3′). Both the forward and reverse 16S primers were tailed with sample-specific PacBio barcode sequences to allow for multiplexed sequencing. The reaction procedure consisted of perform 25 cycles of PCR amplification, with initial denaturation at 95°C for 2 min, followed by 25 cycles of denaturation at 98°C for 10 s, annealing at 55°C for 30 s, and extension at 72°C for 1 min 30 s, and a final step at 72°C for 2 min. The amplicons were quantified, after which the normalized equimolar concentrations of amplicons were pooled and performed Single Molecule Real-Time Sequencing (SMRT) on the PacBio Sequel II platform (Beijing Biomarker Technologies Co., Ltd., Beijing, China). Genomic DNA was randomly broken into 500 bp-10 KB fragments using the library construction Kit (SMRTBELLS PREPKIT 3.0). The fragmented DNA was ligated using the SMRTbell adapter. Unligated adapters and short fragments were removed through magnetic bead screening to enrich the complete target fragments. After repeatedly testing the same target fragment multiple times (passes > 4), the accuracy of Circular Consensus Sequencing (CCS) can reach more than 99%. Using the SMRT Link v8.0 software, the CCS sequence was obtained with minPasses ≥ 5 and minPredictedAccuracy ≥ 0.9 h. After sequencing the samples, CCS sequences were obtained through Barcode recognition. The obtained sequences were preprocessed, including CCS recognition, filtering and removing fragments with higher homology, in order to obtain effective sequences. The valid CCS sequences were clustered using the Usearch (Edgar, 2013) (V10.0.240_i86) with a similarity threshold of 97%, thereby determining the corresponding OTUs for each sample. Taxonomy annotation of the OTUs was performed based on the Naive Bayes classifier in QIIME2 (Bolyen et al., 2019) using the SILVA database (Quast et al., 2013) (release 138.1) with a confidence threshold of 70%, thereby determining the species composition and abundance of each sample at different taxonomic levels (phylum, class, order, family, genus, and species). Alpha was performed to identify the complexity of species diversity of each sample utilizing QIIME2 software. Beta diversity calculations were analyzed by principal coordinate analysis (PCoA) to assess the diversity in samples for species complexity. One-way analysis of variance was used to compare bacterial abundance and diversity.

### Metabolite extraction

For each sample, 100 μL of rumen contents was transferred to a centrifuge tube and mixed with 500 μL of extraction solvent containing an internal standard (methanol: acetonitrile = 1:1, v/v; internal standard concentration 20 mg/L). The mixture was vortexed thoroughly, followed by sonication in an ice-water bath for 10 min using an ultrasonic processor (Xiaomei Ultrasonic Instruments, XM-P22H, China). The samples were then incubated at −20°C for 1 h, after which they were centrifuged at 12,000 rpm (≈13,400 × g) for 15 min at 4°C. A 500-μL aliquot of the supernatant (Want et al., 2010) was transferred to an Eppendorf tube, sealed, and stored at −20°C. All samples were subsequently shipped to Biomarker Technologies (Beijing, China) for mass spectrometry analysis.

### LC–MS/MS analysis

The metabolomics analysis was performed using a liquid chromatography-mass spectrometry (LC–MS) system composed of an Acquity I-Class PLUS ultra-high-performance liquid chromatography (UHPLC) coupled with a Xevo G2-XS QTOF high-resolution mass spectrometer (Waters, United States). The chromatographic separation was achieved on a UPLC HSS T3 column (2.1 × 100 mm, 1.8 μm; Waters, United States). For both positive ion mode (POS) and negative ion mode (NEG), the mobile phase conditions were as follows: mobile phase A was 0.1% formic acid in water, and mobile phase B was 0.1% formic acid in acetonitrile. ESI source parameters were set as: capillary voltage 2,500 V (POS) or −2000 V (NEG); cone voltage 30 V; ion source temperature 100°C; desolvation gas temperature 500°C; backing gas flow rate 50 L/h; desolvation gas flow rate 800 L/h. The injection volume was 1 μL, and a quality control (QC) sample was analyzed after every 10 samples to assess method stability and reproducibility. Data were acquired in MSe mode using acquisition (Jialin et al., 2016) software (V4.2), with a scanning interval of 0.2 s per mass spectrum.

### Data preprocessing and analysis

Raw data collected using MassLynx (V4.2) was processed via Progenesis QI software for peak extraction, alignment, and other preprocessing steps. Compound identification was performed using the Progenesis QI online METLIN database and Biomark’s in-house library, with theoretical fragment annotation and mass deviation constrained within 100 ppm. Principal component analysis (PCA) and Spearman correlation analysis were employed to evaluate intra-group sample reproducibility and quality control (QC) sample consistency. Classification and pathway annotations of identified compounds were retrieved from the Kyoto Encyclopedia of Genes and Genomes (KEGG) (Kanehisa and Goto, 2000), Human Metabolome Database (HMDB) (Wishart et al., 2018), and Lipid Maps Structure Database (LMSD) (Fahy et al., 2007). Fold changes were calculated based on grouping criteria, and T-tests were used to determine statistical significance of *p*-values for individual compounds. Differential metabolites were screened using a combined criterion of fold change >1, *p* < 0.05, and variable importance in the projection (VIP) > 1 from OPLS-DA modeling. Pathway enrichment of differential metabolites was assessed via hypergeometric distribution tests against KEGG pathways.

### Determination and analysis of rumen epithelium transcriptome

Total RNA from the rumen epithelium of Hu sheep was extracted using the Trizol reagent (Life Technologies, California, United States) according to the manufacturer’s instructions. RNA extraction process was as follows: Sample lysis: fifty to 100 mg of rumen epithelial tissue was placed in a pre-cooled mortar, frozen with liquid nitrogen, and ground to a fine powder. The powder was transferred to a centrifuge tube containing 1 mL of TRIzol, then vortexed vigorously or homogenized thoroughly using a tissue homogenizer until the lysate appeared clear and particle-free. RNA separation: two hundred microliters of chloroform were added, and the mixture was shaken for 30 s followed by a 2–3 min incubation at room temperature. After centrifugation at 12,000 × g for 20 min at 4°C, the upper aqueous phase was carefully transferred to a new centrifuge tube. RNA precipitation: Approximately 600 μL of isopropanol was added, and the tube was inverted to mix thoroughly. Following a 10-min incubation at room temperature, the sample was centrifuged at 12,000 × g for 10 min at 4°C, yielding a gel-like or white RNA pellet at the tube bottom. RNA washing: the supernatant was discarded, and 1 mL of pre-cooled 75% ethanol was added to wash the pellet by gentle inversion. After centrifugation at 7,500 × g for 5 min at 4°C, residual ethanol was removed with a pipette, and the pellet was air-dried at room temperature for 5–10 min. RNA dissolution: The pellet was resuspended in 30–50 μL of DEPC-treated water or RNase-free water using a pipette tip, followed by a 5-min incubation at 55–60°C to promote dissolution. RNA concentration and purity were measured using a NanoDrop 2000 spectrophotometer (Thermo Fisher Scientific, Wilmington, DE), and integrity was assessed with the RNA Nano 6,000 Assay Kit on an Agilent Bioanalyzer 2,100 system (Agilent Technologies, CA, United States). Sequencing libraries were generated using Hieff NGS Ultima Dual-mode mRNA Library Prep Kit for Illumina (Yeasen Biotechnology (Shanghai) Co., Ltd.) following manufacturer’s recommendations. After confirming the quality of the libraries, sequencing was performed on the Illumina NovaSeq platform, producing 150 bp paired-end reads. The raw data in fastq format underwent further processing on the bioinformatics analysis platform BMKCloud^1^. Fastp (Chen, 2023) was used by BMKCloud to delete sequences containing joints, sequences containing ploy-N, and low-quality sequences to obtain clean data, and simultaneously calculates Q20, Q30, GC content, and sequence repetition levels. The HISAT2 (Kim et al., 2015) was used to perform rapid and accurate alignment of clean data with the reference genome (Ovis_aries. GCA_017524585.1.) to obtain the localization information of Reads on the reference genome. All downstream analyses relied on high-quality clean data. Effective data were aligned to the reference genome sequence using Hisat2 software (Kim et al., 2019). Gene expression levels were measured using FPKM (Fragments Per Kilobase of transcript per Million fragments mapped). Differential expression analysis between the two groups was conducted using DESeq2, with genes designated as differentially expressed genes (DEGs) if *p* < 0.05 and |FC| ≥ 1.5. GO enrichment analysis and KEGG pathway analysis of DEGs were performed using ClusterProfiler software (V4.2.2). For RNA-seq validation, eight DEGs (PRDX6, FABP5, GNB2L1, GPX1, MAL, NADPH1, PRDX2, IGFBP7) were randomly selected for RT-qPCR analysis, using β-actin as a reference gene. Specific primer sequences are listed in Supplementary Table S1. The reaction conditions for qPCR: initial denaturation at 95°C for 30 s, followed by 40 cycles of 95°C for 5 s and 60°C for 30 s.

### Multi-omics joint analysis

Differentially expressed genes (DEGs) and differentially metabolites with a co-expression trend were mapped to the KEGG pathway, and significantly enriched pathways were screened out. Combined with the functional genera involved in this pathway in the microbiome (combined with literature evidence, genera with clear functional reports in the target pathway were screened out), the “microbiome-metabolism-gene” regulatory axis was constructed.

## Results and analysis

### Growth performance, rumen fermentation characteristics and rumen tissue morphology of Hu sheep

**Figure 1:**
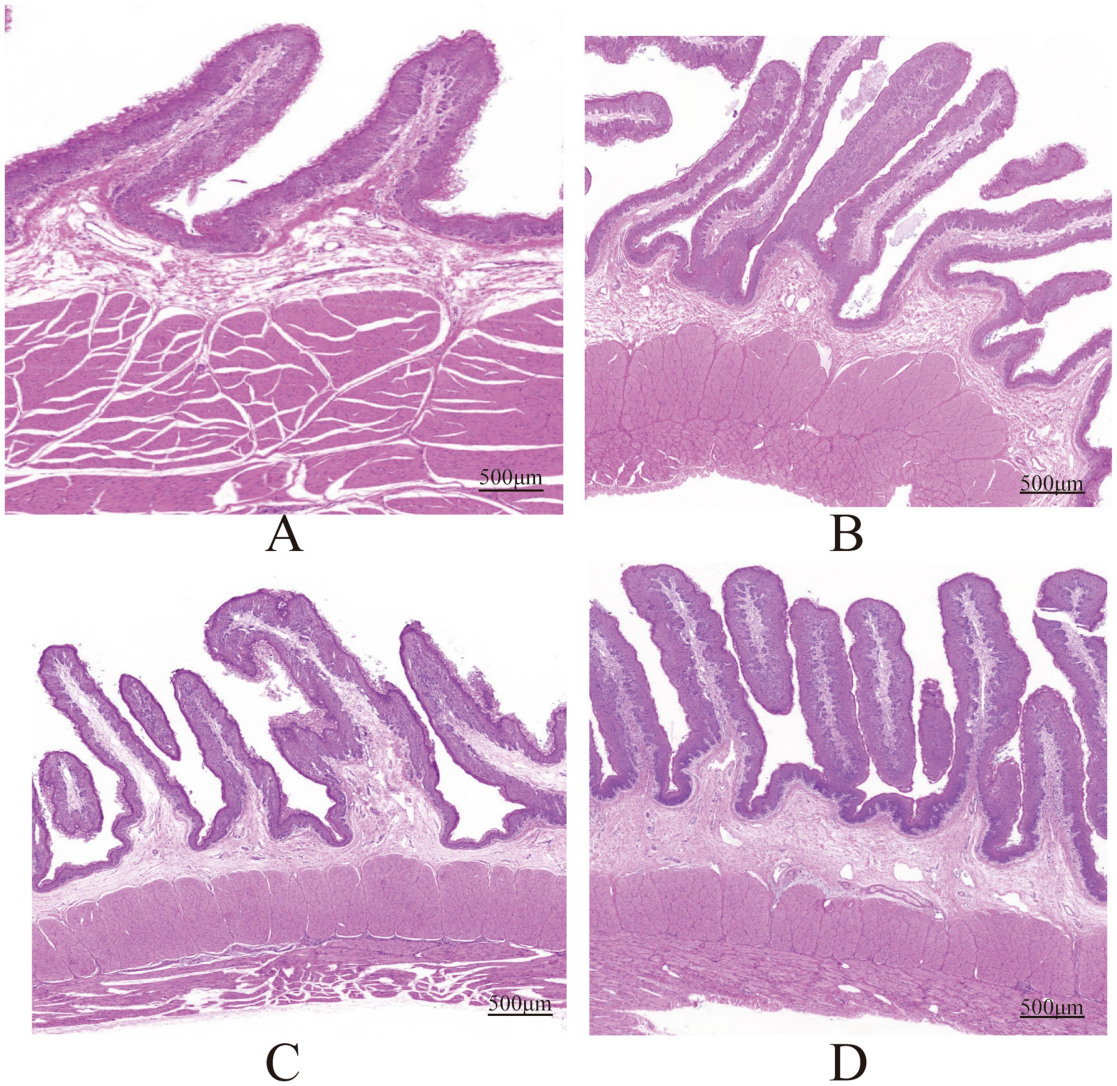
Rumen tissue morphology HE staining in groups (A, B, C, D).

As shown in Table 2, the ADG and ADFI of the roughage groups (B, C, and D) were significantly higher than group A (*p* < 0.05). The ADFI of groups B and C were significantly greater than group A (*p* < 0.05). The FCR of group A was significantly higher than that of group D (*p* < 0.05). The acetic acid content in groups C and D was significantly higher than that in group A (*p* < 0.05). The propionate in groups A and B was significantly higher than group D (*p* < 0.05). group D isobutyric acid, A/P and pH significantly higher than group A (*p* < 0.05). The valeric acid in groups A and B was significantly higher than groups C and D (*p* < 0.05). The TVFA in group A was significantly higher than groups C and D (*p* < 0.05). Additionally, acetate, isobutyric acid, A/P and pH showed a significant linear increase with the increase in FNDF/starch (*p* < 0.05), while propionate, valeric acid and TVFA significantly decreased linearly with the increase in FNDF/starch (*p* < 0.05). The papilla height in groups B and C was significantly greater than group A (*p* < 0.05). The papilla width in group A was significantly greater than group D (*p* < 0.05). Furthermore, papilla height showed a significant quadratic increase with FNDF/starch (*p* < 0.05), while papilla width significantly decreased linearly with FNDF/starch (*p* < 0.05). Rumen tissue section images are shown in Figure 1.

**Table 2:** Analysis of growth performance, rumen fermentation parameters and rumen tissue development.

Items	0.23				SEM	*P*-value		
	A	B	C	D		ANOVA	Linear	Quadratic
Growth performance								
ADG (kg)	0.24^b^	0.30^a^	0.31^a^	0.30^a^	0.01	0.011	0.008	0.028
ADFI (kg)	1.64^c^	2.25^b^	2.48^ab^	2.67^a^	0.09	<0.001	<0.001	0.053
FCR (%)	6.93^c^	7.64^bc^	8.17^ab^	9.07^a^	0.41	0.003	<0.001	0.782
Rumen fermentation parameters								
Acetate (%)	51.32^b^	53.32^ab^	58.02^a^	58.15^a^	1.39	0.026	0.006	0.379
Propionate (%)	32.57^a^	31.06^a^	28.80^ab^	25.21^b^	1.46	0.02	0.003	0.508
Isobutyric acid (%)	2.95^b^	3.01^b^	3.61^ab^	3.83^a^	0.21	0.028	0.005	0.731
Butyrate (%)	5.16	4.94	5.02	4.57	0.4	0.051	0.039	0.056
Isovaleric acid (%)	2.46	2.04	2.2	2.19	0.2	0.594	0.529	0.356
Valeric acid (%)	2.76^a^	2.30^a^	1.52^b^	1.24^b^	0.18	<0.001	<0.001	0.654
TVFA (mmol/^−1^)	117.66^a^	105.68^ab^	97.23^bc^	84.53^c^	4.93	0.002	0.002	0.028
A/P	1.63^c^	1.74b^c^	2.18^ab^	2.31^a^	0.14	0.019	0.003	0.973
pH	6.06^b^	6.07^b^	6.49^ab^	6.70^a^	0.16	0.039	0.007	0.562
Rumen tissue development								
Papilla height (μm)	1990.93^b^	2464.83^a^	2399.15^a^	2301.92^ab^	104.67	0.034	0.095	0.019
Papilla width (μm)	529.02^a^	490.25^ab^	486.44^ab^	420.08^b^	17.76	0.01	0.002	0.492
Basal layer thickness (μm)	1417.97	1296.73	1375.07	1500.47	72.37	0.339	0.361	0.129

In the same row, values with different lowercase letters superscripts mean significant difference (*P* < 0.05), while with the same lowercase letters or no letter superscripts mean no significant difference (*P* > 0.05). Average daily gain (ADG); average daily feed intake (ADFI); feed conversion ratio (FCR); total volatile fatty acids (TVFA); acetic acid/propanoic acid (A/P).

### Rumen microbial composition and microbiota differences

**Figure 2:**
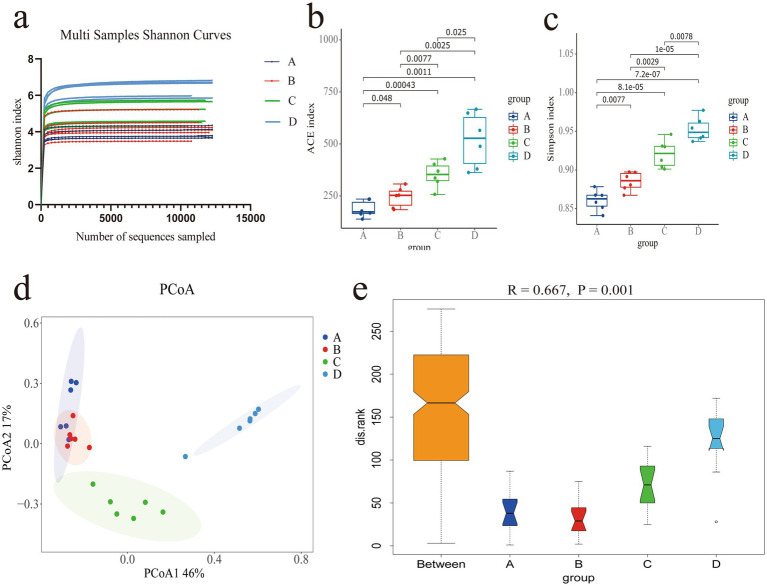
**(a)** Shannon Index curve for the sample. **(b)** ACE index. **(c)** Simpson index. **(d)** PCoA analysis plot. **(e)** Boxplot from Anosim analysis.

Sequencing was conducted on 24 samples and identification was carried out through barcodes. A total of 3.22 million CCS sequences were obtained. Each sample produced at least 11.71 thousand CCS sequences, with an average of 13.44 thousand CCS sequences and an average sequence length of 1,457 bp (Supplementary Table S2). At 1,000 reads, the curve plateaus, indicating that the sequencing data volume is sufficiently large. The number of microbial species does not increase with additional sequencing, suggesting saturation of coverage (Figure 2a). The ACE index (Figure 2b) shows that there are significant differences in species diversity among the groups (*p* < 0.05), and the Simpson index indicates that group D has significantly higher species diversity than groups A, B and C (*p* < 0.05), group B is significantly higher than group A (*p* < 0.05), and group C is significantly higher than group B (*p* < 0.05) (Figure 2c). The PCoA analysis shows high similarity within groups and clear differences between groups. The first and second principal components contribute 46 and 17% to the variance in sample differences, respectively (Figure 2d). Anosim analysis tests the differences between groups, revealing that inter-group differences exceed intra-group differences (*R* = 0.667) and that there are significant differences between groups (*p* = 0.001) (Figure 2e), indicating the meaningfulness of the research groupings.

**Figure 3:**
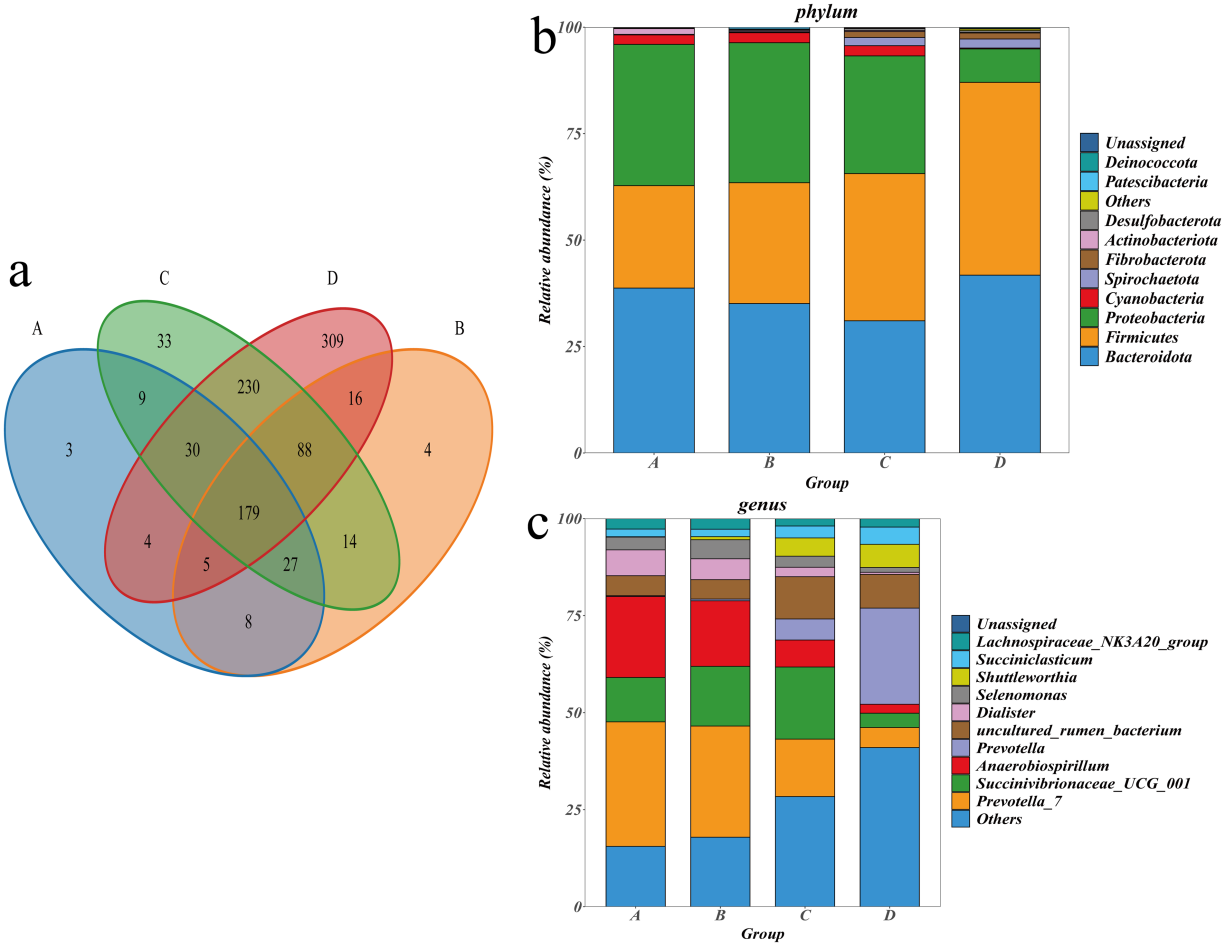
**(a)** Venn diagram of different groups. **(b)** Histogram of the distribution of top 10 species at phylum. **(c)** Histogram of the distribution of top 10 species at genus.

A total of 959 OTUs were obtained by clustering CCS sequences at ≥97.0% similarity level, including 265 OTUs in group A, 341 OTUs in group B, 610 OTUs in group C, and 861 OTUs in group D (Figure 3a). Taxonomically, 22 phyla, 33 classes, 62 orders, 120 families, 245 genera, and 341 species were identified. The top 10 abundant taxa at the phylum and genus levels were annotated as follows: Bacteroidota (36.66%), Firmicutes (33.06%), and Proteobacteria (25.39%) were the dominant phyla (Figure 3b); *Prevotella-7* (20.170%), *Succinivibrionaceae-UCG-001* (12.28%), *Anaerobiospirillum* (11.74%), and *Prevotella* (7.72%) were the dominant genus (Figure 3c); ANOVA was employed to assess the significance of inter-group differences among dominant taxa at the phylum and genus (Supplementary Table S3). At the phylum level, there was no significant difference in Bacteroidota among the groups (*p* > 0.05); Firmicutes in group D was significantly higher than groups A and B (*p* < 0.05); Proteobacteria in Groups A, B, and C was significantly higher than group D (*p* < 0.05); Firmicutes in group D was significantly higher than that in groups A and B (*p* < 0.05). At the genus level, *prevotella-7* in groups A and B was significantly higher than group D (*p* < 0.05). *Prevotella-7* in group A was significantly higher than in group C (*p* < 0.05); *Succinivibrionaceae-UCG-001* in group C was significantly higher than group D (*p* < 0.05); *Anaerobiospirillum* in groups A and B was significantly higher than group D (*p* < 0.05); *Prevotella* in Group D was significantly higher than groups A, B and C (*p* < 0.05); *Ruminococcus* in group D was significantly higher than that in groups A and B (*p* < 0.05).

**Figure 4:**
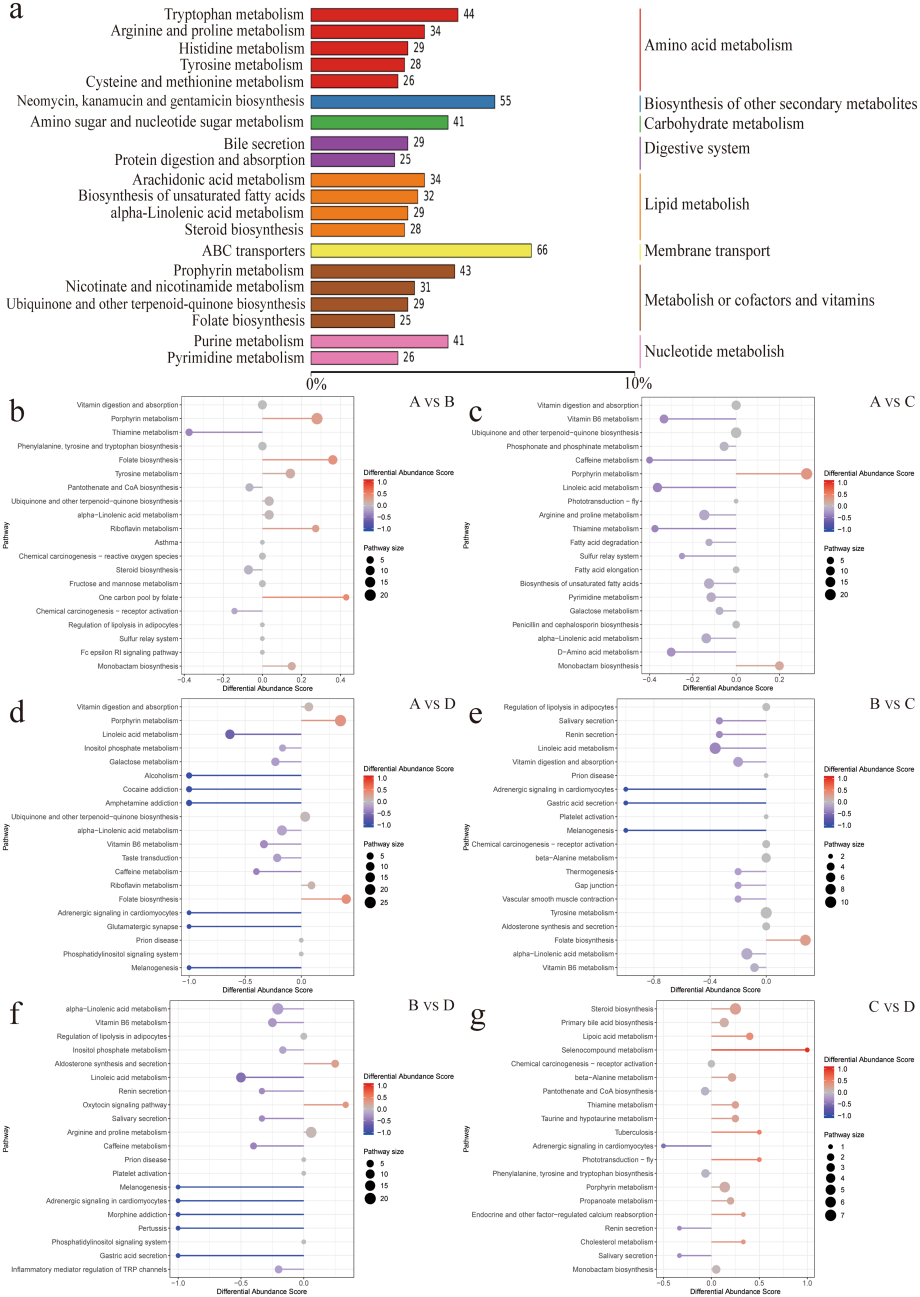
**(a)** Summary of metabolite annotations. **(b–g)** Enriched pathways of differential metabolites.

**Figure 5:**
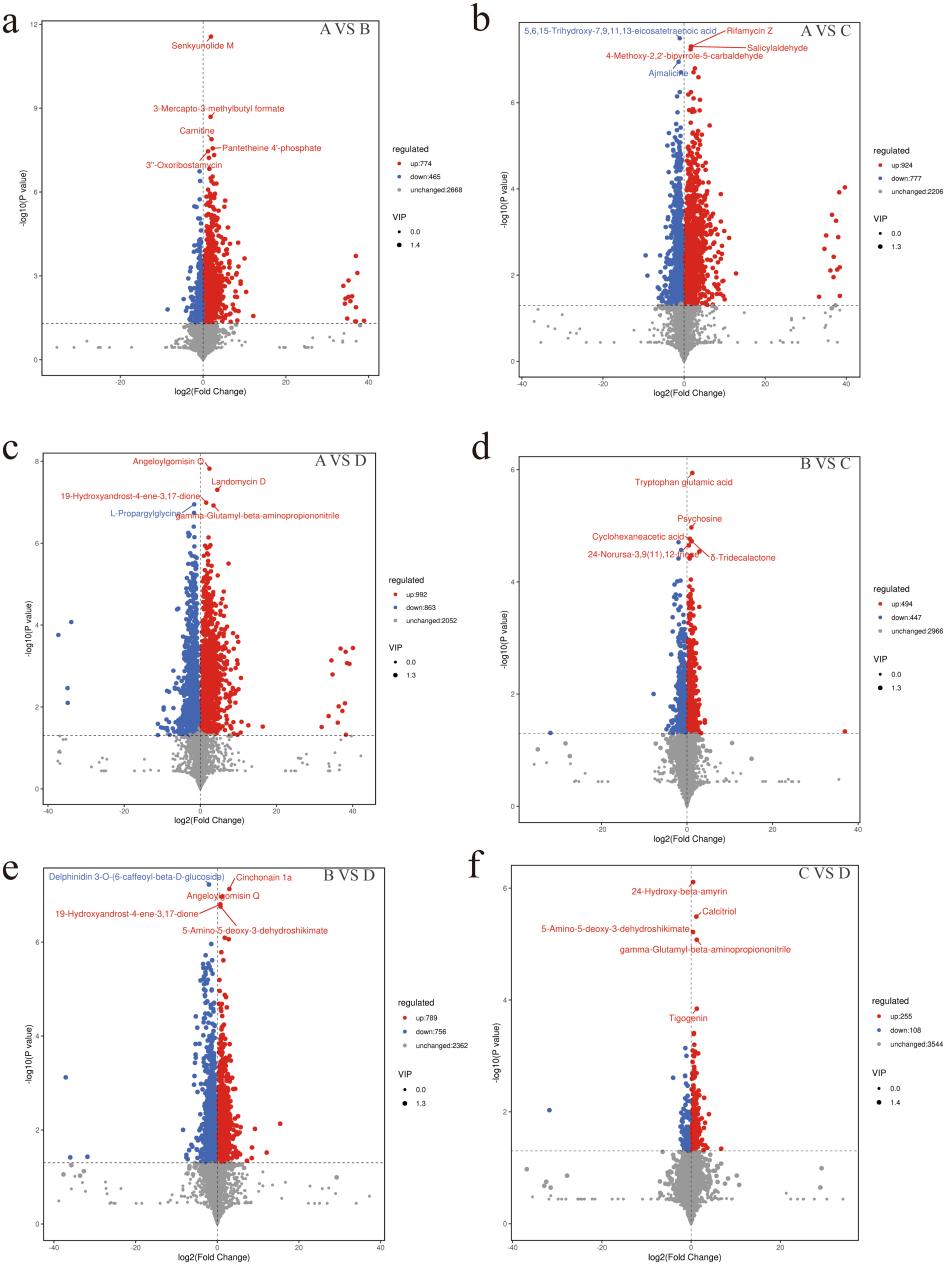
**(a–f)** Volcano plot of differential metabolites.

Metabolite extraction and qualitative and quantitative analysis were performed on 24 rumen contents samples, and 18,810 peaks were identified, among which 3,907 metabolites were annotated (Supplementary Table S4). PCA analysis showed significant differences in metabolites between the groups except for the differences between groups C and D, which were not significant (Supplementary Figure S1). Using the KEGG database, all the identified differential metabolites were annotated and it was found that they were mainly enriched in amino acid metabolism, cofactor and vitamin metabolism, lipid metabolism, biosynthesis of other secondary metabolites, carbohydrate metabolism, digestive system, membrane transport and nucleotide metabolism (Figure 4a). Enrichment analysis of differential metabolites among groups revealed that the main shared pathways included arginine and proline metabolism, biosynthesis of amino acids, histidine metabolism, nicotinate and nicotinamide metabolism, fatty acid metabolism, thiamine metabolism, tryptophan metabolism, tyrosine metabolism, and AMPK signaling pathway (Figures 4b–g). The OPLS-DA score plot revealed R2Y and Q2Y values close to 1 for inter-group comparisons (Supplementary Figures S2a–f, 3a–f), indicating that the evaluation model is stable and reliable, allowing for the selection of differential metabolites. Based on the OPLS-DA results, the selection criteria of |FC| ≥ 1, *p* value <0.05, and VIP ≥ 1 were used to identify the differential metabolites. 774, 924, 992, 494, 789 and 255 upregulated differential metabolites, and 465, 777, 863, 447, 756 and 108 downregulated differential metabolites were identified, respectively, between groups A and B, A and C, A and D, B and C, B and D, and C and D (Figures 5a–f and Supplementary Table S5). Key differential metabolites include thiamine, 3-indole acetamide, 2,2′,3-trihydroxybiphenyl, pyridoxamine phosphate, folic acid, histamine, hercynine, ergothioneine, ergotamine, and niacin (nicotinic acid), etc.

### Differential gene expression and functional enrichment in rumen epithelium

**Figure 6:**
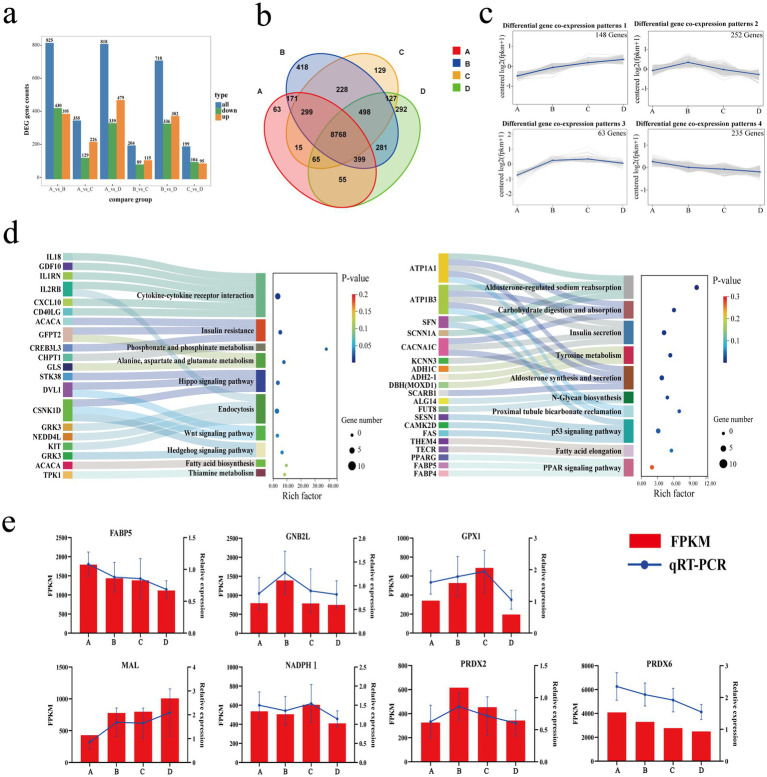
Transcriptome atlas. **(a)** Bar chart of differentially expressed genes. **(b)** Venn diagram of differential gene sets. **(c)** Co-expression pattern of differentially expressed genes. **(d)** Sankey bubble chart. **(e)** Dual *y*-axis chart for qRT-PCR validation of differentially expressed genes.

Twenty-four samples were analyzed for eukaryotic reference transcriptome (RNA-seq), and a total of 159.81 Gb of clean data were obtained. Each sample achieved a clean data output of at least 5.80 Gb, with a Q30 base percentage of 96.66% or higher. The Spearman’s correlation coefficient (*r*) between individuals exceeded 0.65. Clean reads of each sample were aligned with the reference genome of sheep, and the alignment efficiency ranged from 96.49 to 98.61% (Supplementary Table S6). In the comparisons of A vs. B, A vs. C, A vs. D, B vs. C, B vs. D and C vs. D, 825, 355, 818, 204, 718 and 199 differentially expressed genes (DEGs) were identified, respectively, (Figures 6a,b). Trend analysis using K-means clustering revealed that a total of 18 co-expression patterns of differentially expressed genes were discovered (Supplementary Table S7). Among these, four patterns with a higher clustering of differential genes [gene lists for each pattern are available in Supplementary Table S8: patterns 1 (148 genes), patterns 2 (252 genes), patterns 3 (63 genes), and patterns 4 (235 genes)] (Figure 6c) were selected. Notably, a higher k-means value for a gene indicates its stronger representation of the expression pattern associated with this trend. KEGG enrichment analysis (Figure 6d) of DEGs in patterns 1 and 4 revealed that the main KEGG pathways enriched for co-expressed DEGs in pattern 1 included amino acid metabolism, vitamin metabolism, fatty acid synthesis, thiamine metabolism, and signal transduction. In contrast, the main KEGG pathways for pattern 4 included transcription regulation, carbohydrate digestion and absorption, aldosterone synthesis and secretion, and sodium reabsorption regulated by aldosterone, along with signal transduction. The major differential genes in Patterns 1 and 4 are as follows: TPK1, ACACA, IL18, ATP1A1, ATP1B3, FABP4 etc. These genes PABP5, GNB2L, GPX1, MAL, NADPH1, PRDX2 and PRDX6 were randomly selected for qRT-PCR verification. The results indicated that the qRT-PCR data aligned with the trends observed in the RNA-seq data (Figure 6e).

### Multi-omics analysis

**Figure 7:**
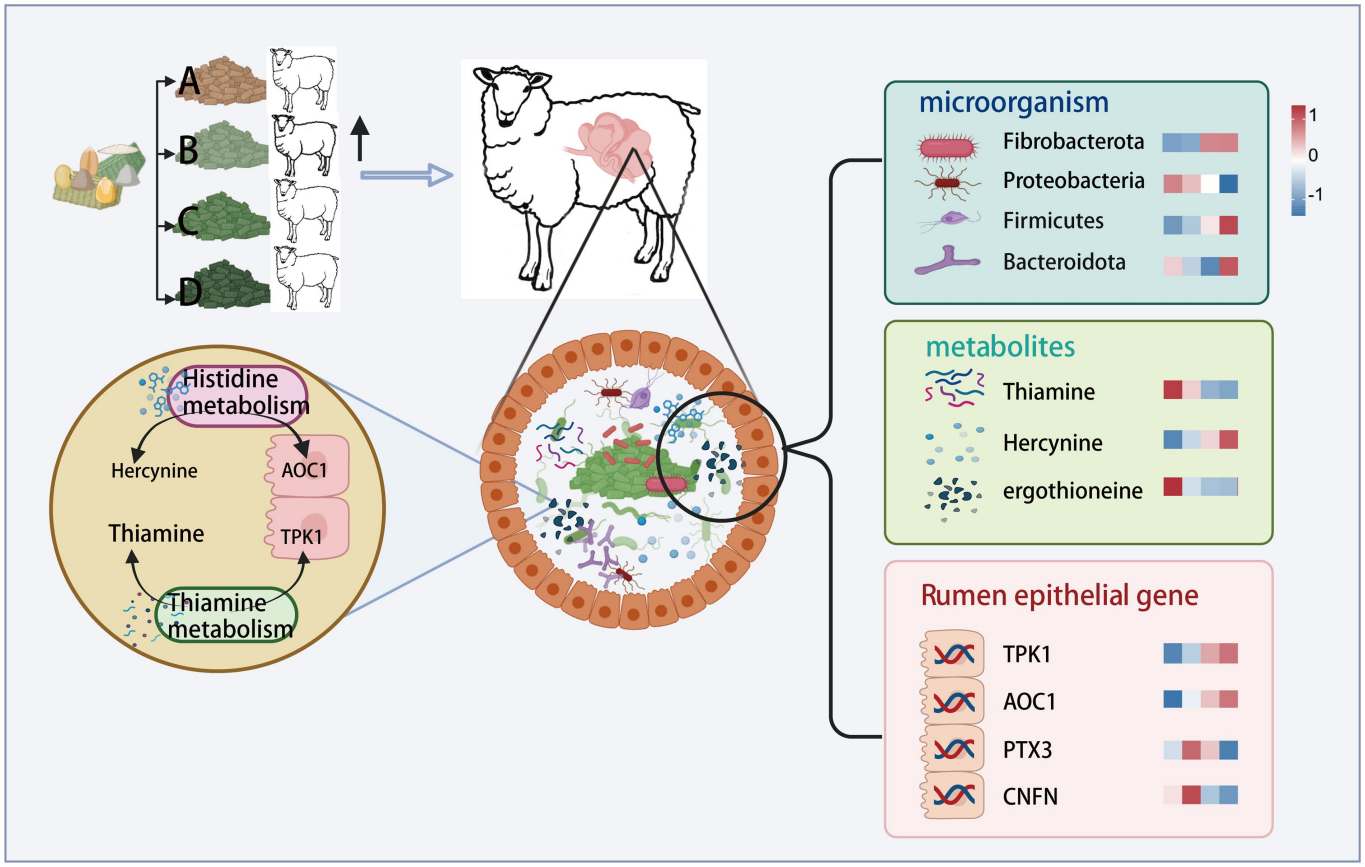
Combined multi-omics analysis.

Multi-omics joint analysis revealed (Figure 7) that during thiamine metabolism, the thiamine content in the rumen contents decreased as the dietary FNDF/starch level increased. Conversely, the expression of the TPK1 gene, which regulates thiamine production in the rumen epithelium, increased with higher dietary FNDF/starch levels. This indicates a negative correlation between TPK1 gene expression in the rumen epithelium and thiamine content in the rumen contents. In the histidine metabolism process, the histamine levels in the rumen contents increased with rising dietary FNDF/starch levels, while the AOC1 gene, associated with histidine metabolism in the rumen epithelium, also showed increased expression with higher dietary FNDF/starch levels.

## Discussion

Growth performance reflects the efficiency of nutrient absorption and utilization in ruminants after nutrient intake. Numerous factors influence animal growth performance, primarily including animal breed, management practices, and environmental conditions. Indicators such as daily weight gain and feed conversion rate can indirectly reflect the alignment between nutrient supply in the diet and the actual needs of the animals. Nutrient intake is the most fundamental requirement for animal growth, while dry matter provides essential nutrients for maintaining health and growth. When nutrient levels meet the needs for sustaining life activities, increased feed intake can enhance nutrient absorption, thereby supplying energy for production activities. In this study, the addition of alfalfa hay and oat hay benefited the average daily weight gain of Hu sheep. The feed intake and feed conversion rate of Hu sheep with an FNDF/starch ratio of 0.23 were lower than those at higher FNDF/starch levels, consistent with findings that increased concentrate levels can enhance the production performance of Tibetan sheep (Liu et al., 2019). The rumen plays a crucial role for ruminants. Numerous studies indicate that feed can alter the VFA concentration in rumen contents, as well as the microbial community and metabolites, particularly the levels of NDF and starch in the feed (Ren et al., 2023; Dias et al., 2017). Therefore, this study integrates the rumen microbiome, metabolome, and epithelial transcriptome to explore the relationships among feed, microbes, metabolites, and the host, while also seeking the optimal balance of FNDF/starch in the diet.

The energy source for ruminants primarily relies on the absorption of short-chain fatty acids. In this study, the contents and ratios of TVFA, acetic acid, and propionic acid will show significant differences. Research indicates that increased concentrations of acetic acid and propionic acid can stimulate the development of rumen epithelium. A higher A/P suggests greater energy utilization efficiency (Zhao et al., 2023). Therefore, it is believed that group B (FNDF/starch = 0.23) and group C (FNDF/starch = 0.56) sheep exhibit higher energy utilization efficiency. Additionally, consistent with this study’s findings, high-starch diets reduce the diversity of rumen contents and decrease the populations of fiber-degrading bacteria such as *Butyrivibrio*, *Ruminococcus* and *Fibrobacter*, while increasing the presence of bacteria like *Prevotella*, *Streptococcus*, *Methanobrevibacter* and *Megasphaera*, which produce propionate and degrade starch (Fernando et al., 2010; Petri et al., 2013). The nutritional level of the diet affects the morphological development of rumen tissues. This study shows that the width of rumen papillae decreases with an increase in FNDF/starch, while the length of rumen papillae develops better with the addition of roughage to the diet. Furthermore, research indicates that high-grain diets can impair rumen development (Xue et al., 2019). Well-developed papillae enhance the digestive capacity of the rumen (Lesmeister et al., 2004). Thus, feeding a diet with an FNDF/starch ratio of 0.23 is more beneficial for the morphological development of rumen tissues. The richness and diversity of microbial communities serve as crucial indicators of rumen digestive capacity. This study’s analysis of rumen microbial diversity indicates that groups C and D exhibit greater microbial diversity, which enhances the stability of the rumen ecosystem and its resilience and adaptability to environmental changes (Zhang Y. K. et al., 2021). Additionally, principal coordinate analysis reveals differences in microbial communities among the various treatment groups. Bacteroidota, Firmicutes, and Proteobacteria account for over 90% of all phyla, consistent with previous findings that these three bacterial groups dominate healthy rumen environments (Chang et al., 2020). Research suggests that improving the Firmicutes/Bacteroidota (F/B) ratio in the gut can enhance feed efficiency and promote energy absorption in hosts (Sim et al., 2022). In this study, group C shows a higher F/B ratio, indicating improved feed efficiency. Studies have demonstrated that Bacteroidetes and Firmicutes are the most preponderant phyla in the gastrointestinal microbiota of mammals (Pu et al., 2020). Specifically, Bacteroidetes play a crucial role in the degradation of non-fibrous components, while the Firmicutes phylum is primarily associated with fiber degradation (Jami and Mizrahi, 2012). Consistently, the present study identified Bacteroidetes and Firmicutes as the dominant phyla. Notably, when the FNDF/starch ratio reached 1.12, the relative abundance of Firmicutes exhibited a significant upward trend, providing further evidence for the involvement of Firmicutes in fiber degradation processes. Additionally, previous studies have reported that the abundance of Proteobacteria increases with the elevation of dietary NFC/NDF levels (Li et al., 2023), which is generally consistent with the finding of this study: the abundance of Proteobacteria decreases as the dietary FNDF/starch ratio increases. At the genus level, the predominant bacteria are *Prevotella* and *Prevotella_7*, aligning with earlier 16S rRNA studies (Wang et al., 2022; Savin et al., 2022). Interestingly, in this study, the relative abundance of *Prevotella* and *Prevotella_7* exhibits opposing trends with increasing FNDF/starch ratios, while the composition of structural and non-structural carbohydrates in the diet also shows contrasting trends. This might indicate that *Prevotella* and *Prevotella_7, respectively,* use structural and unstructural carbohydrates in the diet as the main degradation substrates. *Ruminococcus* belongs to the Firmicutes and was known to degrade cellulose in the rumen (Whon et al., 2021). The abundance of *Ruminococcus* in different NFC/NDF diets varied, resulting in changes in fiber digestibility and the formation of short-chain fatty acids (SCFAs) (Chen et al., 2024). In this study, the abundance of *Ruminococcus* increased with the increase of dietary FNDF/starch levels, indicating that a high-fiber diet might increase the colonization of cellulose-decomposing bacteria.

Metabolite analysis reveals that feeding different FNDF/starch diets to Hu sheep resulted in notable differences in rumen metabolites. For example, in the metabolic pathway (ko01100), with the increase of FNDF/starch, the level of 2,2′, 3-trihydroxybiphenyl was detected to increase significantly (there was no difference between group C and group D). Under the action of estradiol ring cleavage dioxygenase, 2,2′,3-trihydroxybiphenyl is converted into 2-hydroxy-6-(2-hydroxyphenyl)-6-oxo-2,4-hexadienoic acid, which undergoes cyclization and hydrolysis to yield salicylic acid (Kohler et al., 1993). Salicylic acid inhibits inflammatory responses by regulating the gut microbiota (for example, reducing the abundance of Prevotella) and reduces methane production *in vitro* through mechanisms related to its lipophilic molecular structure (Nørskov et al., 2023; Luo et al., 2022). Additionally, this study identified differential metabolites involved in the digestion and absorption of vitamins, with increased levels of pyridoxamine phosphate and folic acid, and decreased levels of Thiamine and Vitamin D3 as FNDF/starch increased. Group A showed significantly higher pantothenic acid levels compared to other groups, consistent with numerous studies indicating that feed levels are a major factor affecting the differences in metabolites related to vitamin digestion and absorption in the rumen (Tafaj et al., 2006; Ragaller et al., 2011; La et al., 2019). The digestion and absorption of vitamins directly influence the biosynthesis and catabolism of amino acids, thereby affecting the operation of the digestive system and leading to variations in growth performance (Miret and Munné-Bosch, 2014). Histamine and hercynine were identified as differential metabolites in histidine metabolism. In this study, histamine levels gradually decreased while hercynine levels increased with higher FNDF/starch in the diet. The results also confirmed the presence of high concentrations of histamine in the rumen fluid of animals fed high-concentrate diets (Sun et al., 2017). Histamine can induce inflammatory responses in rumen epithelial cells (Wang et al., 2013), while hercynine is a metabolite of the strong antioxidant ergothioneine (Ando and Morimitsu, 2021), suggesting that increased FNDF/starch in the diet may enhance the body’s antioxidant capacity. The study posits that ergotamine can impair the immune system, affect gastrointestinal activity (e.g., gut motility and emptying), and influence hormones that regulate feeding (e.g., insulin, leptin), leading to reduced feed intake (McLeay and Smith, 2006). In this study, ergotamine significantly increased with higher FNDF/starch in the diet, particularly in group D, where the average abundance of ergotamine was three times that of group B and twice that of group C, which is detrimental to rumen health. However, appropriate levels of ergotamine do not adversely affect animal health, nutrition, or growth performance (Poole et al., 2009). In this study, niacin levels in groups A and B were significantly higher than in groups C and D, indicating that excessive FNDF/starch is detrimental to the health and development of rumen epithelium in ruminants.

In order to understand the effects of different FNDF/starch diets on the expression of host genes, multiple differentially expressed genes (DEGs) were identified through transcriptome sequencing in this study. Among them, the PTX3 gene serves as a biomarker and evaluation factor in immunity, with its encoded protein playing a corresponding immune role upon pathogen invasion (Jaillon et al., 2007; Suliman et al., 2008). The expression of PTX3 decreases with increasing FNDF/starch in the diet, regulating proteins in the rumen and thus maintaining normal rumen function. Research indicates a significant correlation between the relative expression of the TPK1 gene and thiamine levels (Sambon et al., 2022). In this study, the upregulation of TPK1 expression may occur due to the decrease in thiamine content in the rumen as the FNDF/starch ratio in the diet increases. This reduction in thiamine availability prompts the host to enhance TPK1 expression through feedback regulation mechanisms, facilitating thiamine production within the body (Pavlova et al., 2021). FABP4 (fatty acid-binding protein 4) and FABP5 (fatty acid-binding protein 5) encode fatty acid-binding proteins found in adipocytes. They bind to long-chain fatty acids (FAs) and participate in their uptake, transport, and metabolism (Thompson et al., 2018; Rosa-Velazquez et al., 2022; Li et al., 2020). In this study, as the FNDF/starch ratio in the diet increases, the expression of the FABP4 gene in the rumen tissue of Hu sheep gradually decreases. This finding suggests that the mobilization and metabolism of long-chain FAs in the rumen decline with increasing FNDF/starch in the diet. Conversely, the expression levels of ACACA (acetyl-coenzyme A carboxylase-*α*) and TPK1 increase with the FNDF/starch ratio. ACACA is involved in the synthesis of fatty acids from acetate, likely due to elevated acetate concentrations in the rumen, which enhances the expression of genes that utilize acetate (Zhang et al., 2023). Although the mechanisms differ from those of FABP4, the outcomes align. ATP1A1 and ATP1B3 encode the α1 and β3 subunits of the Na+/K+-ATPase, respectively. Both are widely expressed across various tissues and function as transmembrane proteins on the cell membrane. They establish a high K+/low Na+ gradient, crucial for maintaining transmembrane potential, cellular homeostasis, physiological activities, and normal metabolism (Dieho et al., 2017). Studies show a positive correlation between ATP1A1 expression and VFAs concentrations, with hay and concentrate feeding affecting ATP1A1 expression. This finding is consistent with the results of the current study (Metzler-Zebeli et al., 2013). Furthermore, two significant upregulated genes, GCNT3 and SLC9A3, were identified. Research indicates that SLC9A3 plays a role in the rumen by transporting sodium into cells and protons into the rumen, thereby helping to maintain epithelial homeostasis (Beckett et al., 2021; Connor et al., 2010). In this study, the expression of SLC9A3 in the roughage-added groups (B, C, and D) was significantly upregulated compared to the non-roughage-added group, suggesting that the addition of roughage in the diet benefits rumen epithelial stability, independent of FNDF/starch levels. The protein encoded by GCNT3 is associated with tissue integrity (Jiang et al., 2023). In this study, the expression of the GCNT3 gene was upregulated in the roughage-added groups compared to the non-roughage-added group, indicating that adding roughage to the diet may enhance the integrity of the rumen epithelium. These findings underscore the importance of dietary composition in modulating gene expression related to rumen health. The upregulation of both SLC9A3 and GCNT3 in response to roughage supplementation suggests a potential mechanism through which dietary strategies can be employed to optimize rumen function and overall animal health. Further investigations are warranted to elucidate the precise pathways involved and to explore the implications of these findings for improving feed efficiency and animal performance. Additionally, the interaction between FNDF/starch levels and roughage types could provide valuable insights into formulating diets that maximize the benefits of roughage while minimizing potential negative effects associated with high-starch diets. Future research should also consider the long-term effects of these dietary modifications on rumen morphology and microbial populations, as these factors are critical for sustaining optimal digestive health in ruminants.

## Conclusion

This study explored the effects of different FNDF/starch ratios in diets on the growth performance, rumen microbiota, metabolome and epithelial transcriptome of Hu sheep. The results showed that adding alfalfa hay and oat hay improved the average daily weight gain of Hu sheep. A diet with an FNDF/starch ratio of 0.23 was more beneficial to the morphological development of rumen tissues, while groups C and D (FNDF/starch = 0.56 and 1.12) exhibited greater microbial diversity, enhancing the stability of the rumen ecosystem. Bacteroidota, Firmicutes, and Proteobacteria were the dominant phyla, accounting for over 90% of the microbial community, consistent with previous studies. The Firmicutes/Bacteroidota ratio was higher in group C, indicating improved feed efficiency. Notably, the relative abundance of Firmicutes significantly increased when the FNDF/starch ratio reached 1.12, suggesting their crucial role in fiber degradation. At the genus level, *Prevotella* and *Prevotella_7* were predominant, with contrasting abundance trends as FNDF/starch increased, implying their differential utilization of structural and non-structural carbohydrates. The abundance of *Ruminococcus*, a cellulose-degrading bacterium, increased with higher FNDF/starch ratios, indicating enhanced colonization of fiber-degrading bacteria in high-fiber diets. Metabolite analysis revealed that different FNDF/starch ratios affected the levels of short-chain fatty acids, vitamins, and antioxidant-related substances. Transcriptome sequencing identified differentially expressed genes related to immunity, energy metabolism, and rumen epithelial integrity, such as PTX3, TPK1, and FABP4. The study concluded that the FNDF/starch ratio in the diet significantly influenced the growth performance and rumen health of Hu sheep by regulating the rumen microbiota, metabolites and host gene expression.

## Data Availability

The data presented in this study are deposited in the Sequence Read Archive (SRA) of the National Center for Biotechnology Information (NCBI), with accession numbers PRJNA1194784 (SRA: 41399001-41399024) and PRJNA1274619 (SRA: 44080878-44080901) for transcriptome and microbial data, respectively. Additionally, the metabolomic data are deposited in the Genome Sequence Archive (GSA) under accession number PRJCA041519.

## Footnotes

^1^ www.biocloud.net
