# Supplementary figures and images for: Multi-omics reveals effects of diet FNDF/starch level on growth performance and rumen development of Hu sheep
Source: Front Microbiol. 2025 Aug 5;16:1601950. doi: 10.3389/fmicb.2025.1601950 (PMC12361173; doi:10.3389/fmicb.2025.1601950)

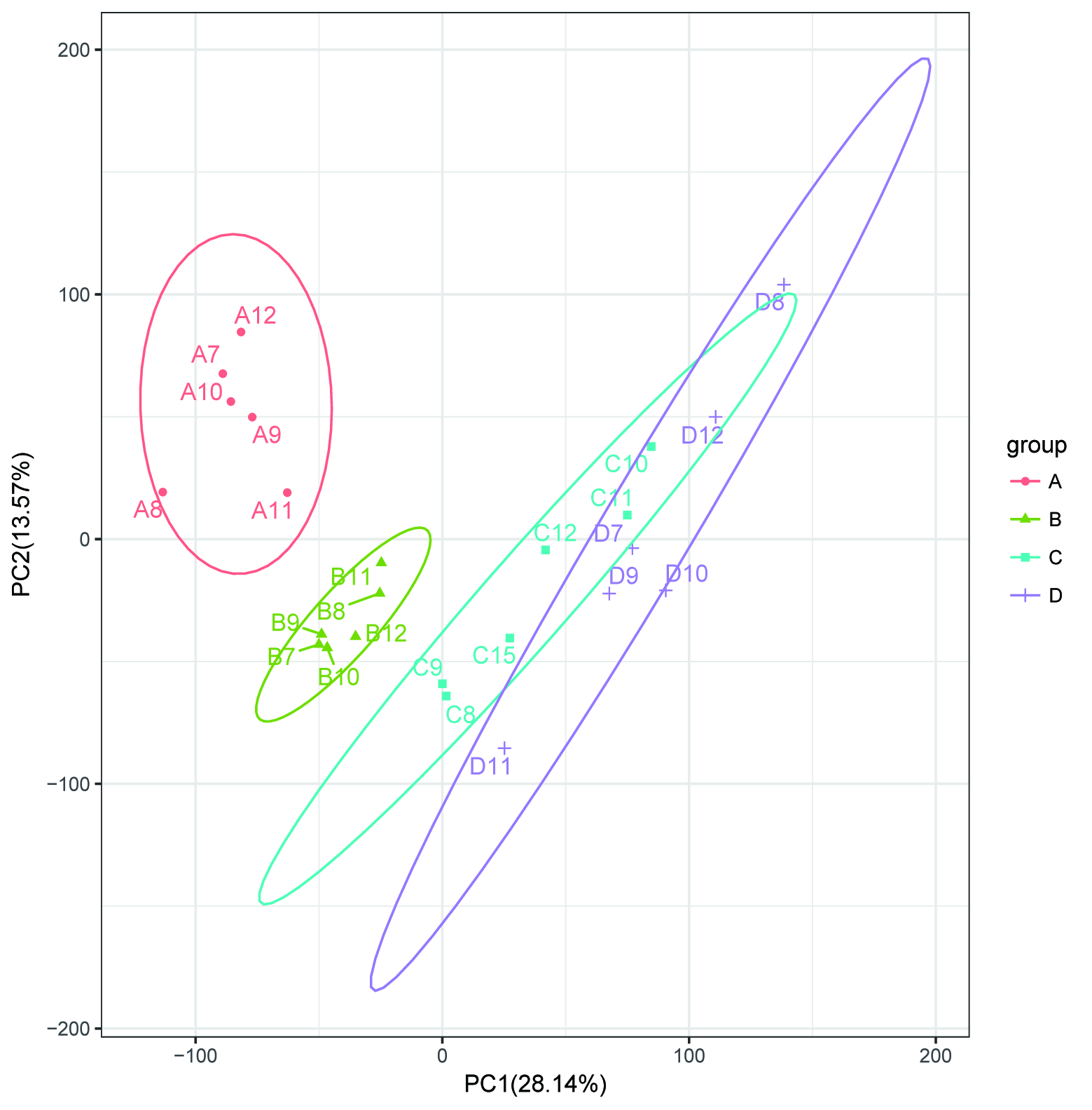

Supplement: Supplementary file 9 [file Image_1.tif]

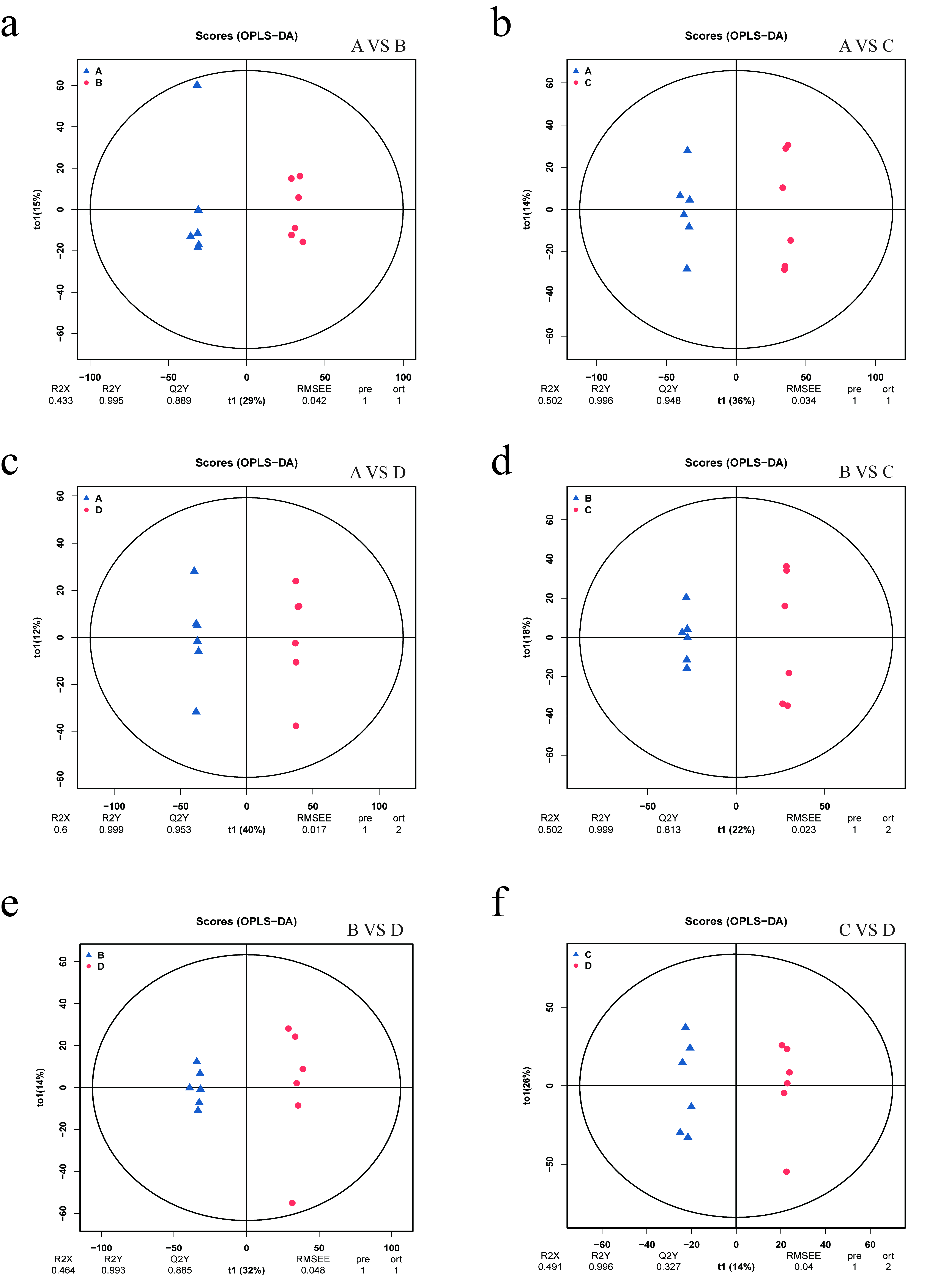

Supplement: Supplementary file 10 [file Image_2.tif]
